# Specific Involvement of Pilus Type 2a in Biofilm Formation in Group B *Streptococcus*

**DOI:** 10.1371/journal.pone.0009216

**Published:** 2010-02-15

**Authors:** Cira Daniela Rinaudo, Roberto Rosini, Cesira L. Galeotti, Francesco Berti, Francesca Necchi, Valerio Reguzzi, Claudia Ghezzo, John Laird Telford, Guido Grandi, Domenico Maione

**Affiliations:** Novartis Vaccines and Diagnostics, Siena, Italy; National Institute of Allergy and Infectious Diseases, National Institutes of Health, United States of America

## Abstract

*Streptococcus agalactiae* is the primary colonizer of the anogenital mucosa of up to 30% of healthy women and can infect newborns during delivery and cause severe sepsis and meningitis. Persistent colonization usually involves the formation of biofilm and increasing evidences indicate that in pathogenic streptococci biofilm formation is mediated by pili. Recently, we have characterized pili distribution and conservation in 289 GBS clinical isolates and we have shown that GBS has three pilus types, 1, 2a and 2b encoded by three corresponding pilus islands, and that each strain carries one or two islands. Here we have investigated the capacity of these strains to form biofilms. We have found that most of the biofilm-formers carry pilus 2a, and using insertion and deletion mutants we have confirmed that pilus type 2a, but not pilus types 1 and 2b, confers biofilm-forming phenotype. We also show that deletion of the major ancillary protein of type 2a did not impair biofilm formation while the inactivation of the other ancillary protein and of the backbone protein completely abolished this phenotype. Furthermore, antibodies raised against pilus components inhibited bacterial adherence to solid surfaces, offering new strategies to prevent GBS infection by targeting bacteria during their initial attachment to host epithelial cells.

## Introduction

A number of studies have revealed that many bacteria and fungi exist predominantly as surface-attached multicellular communities, commonly referred to as biofilms, embedded in bacterial-derived extracellular matrix typically containing exopolysaccharides, proteins and nucleic acids. Biofilm development is a multistep process, in which component cells acquire phenotypes that are distinct from their planktonic (or free-floating) counterparts, and is considered critical for numerous bacterial infections [1]. To switch from the planktonic to sessile lifestyle bacteria have to undergo a series of genetically regulated events and several studies have indicated that biofilm formation proceeds through a five-stage developmental program. A loose or transient association with a surface, followed by robust adhesion, generally identifies stages one and two. Stages three and four involve the aggregation of cells into microcolonies and subsequent growth and maturation. Stage five is characterized by a return to transient motility, where biofilm cells are sloughed off or shed [2]. The study of bacteria residing in biofilms as an interactive community rather than free-living planktonic cells has recently gained much attention as a result of a recent estimate by the Centers for Disease Control and Prevention that more than 65% of human bacterial infections involve biofilms [3]. Many species of streptococci are known to form biofilms [4].

The complex pathway leading to biofilm development in different species of microorganisms involves the contribution of both environmental conditions and genetic factors. Numerous genes or factors have been identified as being essential or required for biofilm formation [5]. Such genes include those that regulate surface-exposed proteins, appendages such as pili or fimbriae, and extracellular polymeric substance (EPS) matrix materials. Pili seem to play a key role in adhesion and attachment to host cells both in Gram-negative and Gram-positive pathogens. Their involvement in the transition from planktonic growth to a surface-attached multicellular community has also been demonstrated in many studies [6]. For instance, fimbriae and pili have been implicated in mediating coaggregation and biofilm formation in Actinomycetes, Enterococci and Streptococci [7], [8], [9], [10], [11].

*Streptococcus agalactiae* (Group B Streptococcus [GBS]) is a Gram-positive pathogen that causes severe invasive neonatal infections, such as pneumonia, septicemia and meningitis [12]. This microorganism is also responsible for significant morbidity in pregnant women and the elderly, and is a serious cause of mortality in immunocompromised adults [13]. However, *S. agalactiae* is primarily a commensal opportunistic organism, colonizing the gastrointestinal and genitourinary tracts of up to 30% of healthy adults. This asymptomatic colonization is known to precede the majority of cases of neonatal invasive infection, acquired during delivery by direct mother-to-baby transmission of the pathogen [14]. GBS can also colonize the mammary glands of ruminants, where it is able to survive for long periods, causing clinical and sub-clinical mastitis [15], [16], [17]. GBS strains have been isolated, in association with other known biofilm-forming bacteria, from biofilms on intrauterine devices [4], [18] and the ability to form biofilm in microtiter plates has been recently reported for the GBS strain NEM316 [9] and for GBS clinical isolates from North India [19].

Three different types of pili have been characterized in GBS as potential virulence factors and promising vaccine candidates due to their ability to induce protective immunity in animal models [20], [21], [22], [23]. Such structures have also been implicated in mediating attachment to human epithelial cells [9], [24], [25], in the adhesion and invasion of brain microvascular endothelial cells [26] and in promoting transepithelial migration [27]. The genes involved in the synthesis and assembly of the three GBS pili are clustered in characteristic genomic loci (named PI-1, PI-2a and PI-2b), each encoding three structural proteins containing a LPXTG motif and two dedicated subfamily C sortases (SrtC) involved in covalent polymerization of the subunits [22].

In the present study, we provide evidence that GBS human isolates can form biofilms on abiotic and biotic surfaces and that type 2a pili are involved in biofilm formation. By analyzing a wide panel of GBS clinical isolates, previously screened for the presence of pili [23], we observed a statistically significant correlation between expression of pilus type 2a and the ability to form biofilm *in vitro*. The specific involvement of pilus type 2a, but not pilus types 1 and 2b in biofilm development is here confirmed by gene deletion and complementation analysis. Moreover, we present data showing that the minor ancillary protein of pilus 2a, whose role in pilus anchoring to the cell wall, a process mediated by the constitutive sortase A has been recently demonstrated [28], plays a key role in biofilm development. Finally, we also show that antibodies directed against surface-exposed pilus 2a proteins are effective in interfering with the initial steps of biofilm formation, suggesting a potential novel strategy to inhibit bacterial adhesion and to prevent implant-associated infections.

## Results

### Group B *Streptococcus* Strain 515 Can Adhere to Polystyrene Plates in the Presence of Glucose and Sucrose

**Figure 1:**
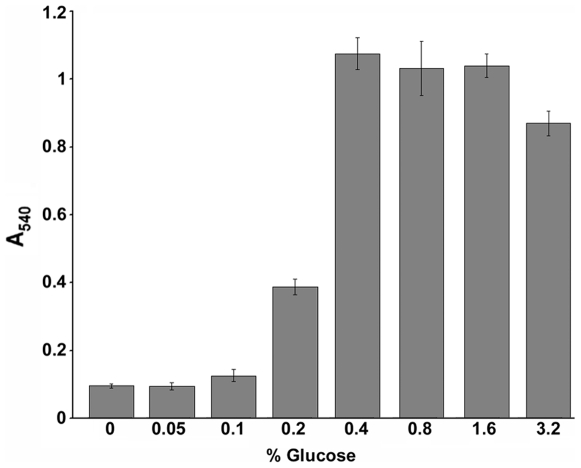
Influence of glucose on biofilm formation in GBS strain 515. Determination of biofilm formation by crystal violet (CV) assay of GBS strain 515 grown in THB supplemented with different glucose concentrations in 96-well polystyrene plates under static conditions at 37°C, 5% CO_2_ for 18 hours. Crystal violet stained, surface-attached cells were quantified by solubilizing the dye in 30% acetic acid and by measuring the absorbance at 540 nm. The mean values of three independent experiments and standard deviation are shown.

To assess the capacity of GBS to form biofilms, we first analyzed whether the serotype Ia strain 515 [29], expressing only pilus type 2a and previously used for pili genetic studies [22], was able to adhere to polystyrene surfaces, using the well-established biofilm assay described by O'Toole and Kolter [30]. Bacteria were grown in tissue culture plates and biofilm formation was detected by crystal violet (CV) staining, followed by dye solubilization with acetic acid. Since growth medium composition influences the capacity to form biofilm in other streptococci [31], [32], GBS strain 515 was cultured under static conditions in THB, a standard streptococcal growth medium, and in THB supplemented with increasing concentrations of glucose and biofilm formation was assessed after 18 hours of growth. While the presence or absence of glucose did not affect growth rates when bacteria were cultured in suspension (data not shown), only in the presence of glucose concentrations ≥0.4%, surface adhesion and proliferation was observed (Figure 1). Similar results were obtained using sucrose instead of glucose (data not shown).

### High Surface Exposure of Pilus Type 2a Strongly Correlates with Biofilm Formation

**Figure 2:**
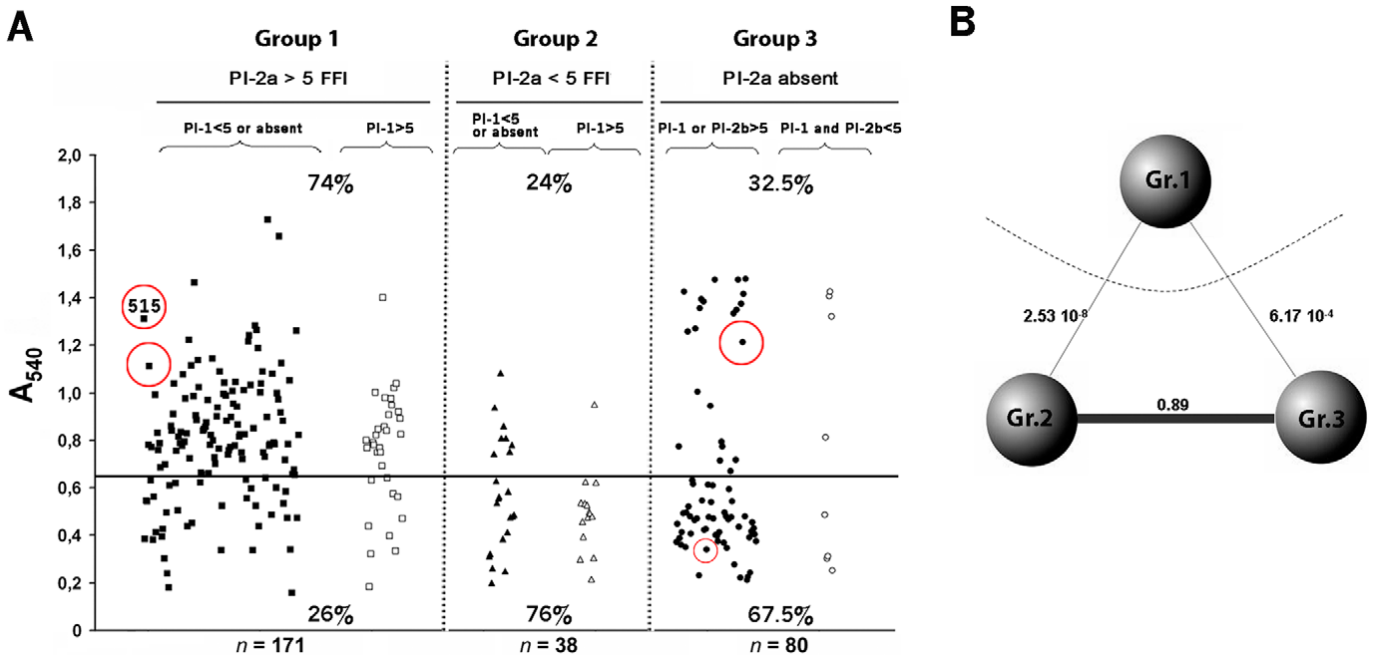
Correlation between surface exposure of pilus type 2a and biofilm formation. (A) Determination by crystal violet (CV) assay of biofilm formation of 289 GBS clinical isolates grown in 96-well polystyrene plates in THB supplemented with 1% glucose at 37°C for 18 h. Adherent bacteria were stained with crystal violet (CV) and quantified by measuring the absorbance at 540 nm. The data represent the mean values of three experiments. Standard deviations (not shown) ranged from OD_540_ values of 0.02 to 0.1. Strains were distributed into 3 groups according to the level of expression of pilus type 2a (PI-2a, Pilus Island 2a), measured by flow cytometry as the Fluorescence Fold Increase (FFI) of cells stained with anti-pilus 2a sera over cells stained with pre-immune sera. Group 1 (High pilus expression: PI-2a >5 FFI) including 171 strains; Group 2 (intermediate or low pilus expression: PI-2a <5 FFI) including 38 strains and Group 3, in which the PI-2a was absent, including 80 strains. Group 1 or Group 2 were also subdivided in strains expressing high levels of pilus type 1 (PI-1>5) and strains carrying only pilus 2a (PI-1 absent) or expressing pilus type 1 below the threshold level (PI-1<5). Group 3 was subdivided into strains expressing either PI-1 or PI-2b at high levels (FFI >5) and strains in which also PI-1 and PI-2b poorly expressed (FFI <5). The horizontal bar intersecting the value of absorbance 0.65 represents the cut-off established to discriminate between high-biofilm forming strains and low-biofilm forming strains. The cut-off was calculated as the value equidistant from the median of absorbance of groups 1, 2 and 3. The percentages are calculated considering the number of strains over or below the cut-off value. Circles indicates strains used for SEM analysis shown in Figure 3. (B) Non parametric multi-comparison statistical analysis. The Kruskal-Wallis test was applied to test the equality of population median among the groups and confirmed that the three groups did not belong to the same population (p-value = 1.742 e^−08^). The Behrens-Fisher test was applied to distinguish which of the groups were significantly different. Each of the lines connecting two groups is proportional to their comparison p-value and confirms a strong relation between Gr.2 and Gr.3, while the Gr.1 was found to be very different from the others.

To investigate if the capacity to adhere to polystyrene plates observed in strain 515 was a general property of all GBS strains, we next analyzed the biofilm-forming capacity of a wide panel of GBS clinical isolates (a total of 289 strains), obtained from infants and adults with invasive diseases. These strains had been previously shown to express at least one of the three pilus types identified in GBS [23]. For the screening of the entire panel of isolates, bacteria were grown in 96 well microtiter plates in THB supplemented with 1% glucose and biofilm formation was assayed after 18 hours of static growth. A summary of the results is given in Figure 2 where bacteria are grouped on the basis of the expression level of pilus type 2a, as judged by FACS analysis using anti-pilus 2a antibodies [23] (group 1: high expression, group 2: low expression, group 3: expression not detectable). As shown in Figure 2A, 74% of the strains expressing pilus 2a at high levels were capable of forming biofilm, whereas low and no expression of pilus 2a resulted in a low capacity of growing in biofilm (24% and 32.5% of the strains, respectively). A non parametric multi-comparison analysis, using the Behrens-Fisher test, confirmed that the group of strains expressing high levels of pilus 2a (Group 1) was well distinguishable from the two groups of strains in which pilus 2a was either poorly expressed (Group 2) or not expressed (Group 3) (Figure 2B).

**Figure 3:**
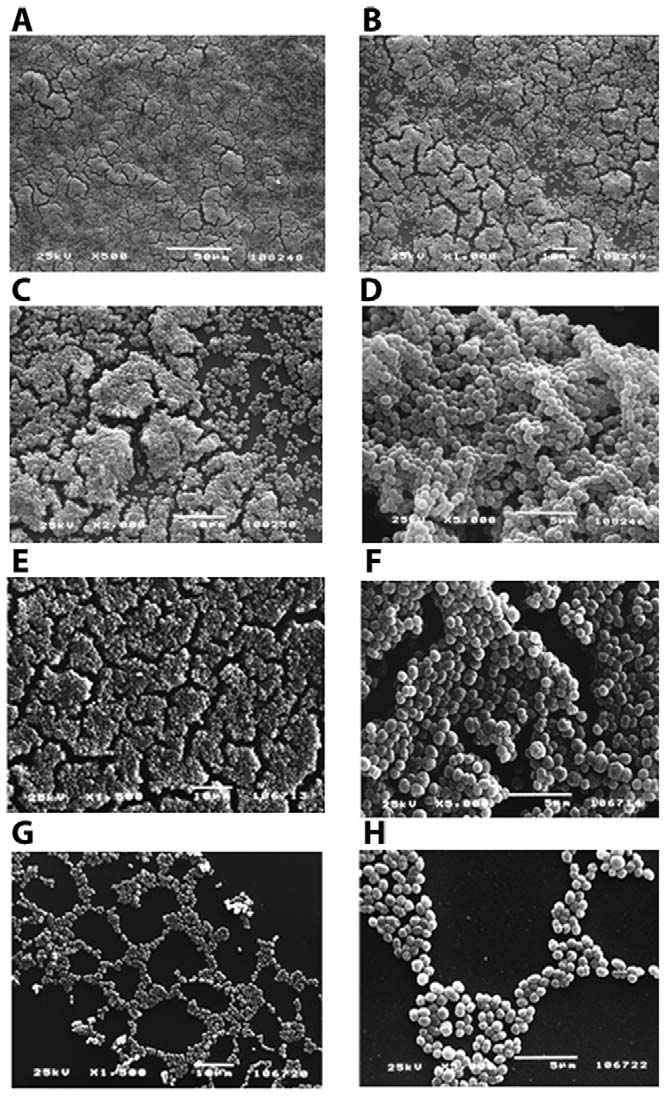
Morphological analysis of GBS biofilms by Scanning Electron Microscopy. Representative SEM images of three different GBS high-biofilm former strains (A–F) and of a low-biofilm former strain (G–H). Bacteria were grown on uncoated glass coverslips for 72 h in static conditions. (A and B) Strain CHR-021, serotype Ia with magnification ×500 (A) and ×100 (B). (C and D) Strain ABC020018145, serotype II with magnification ×2000 (C) and ×5000 (D). (E and F) Strain 515, serotype Ia with magnification ×1500 (E) and ×5000 (F). (G and H) Strain CHR-019, serotype III with magnification ×1500 (G) and ×5000 (H).

Scanning electron microscopy (SEM) analysis of three different strains, GBS strain 515 and two randomly selected strains from the pool of high biofilm formers (circled in Figure 2A) confirmed that these strains grew on the surface in three-dimensional, multilayered cell aggregates. At higher magnification the presence of cell aggregates was apparent (Figure 3A–F). By contrast, microscopy analysis of the surface of the well where a poor biofilm-forming strain was grown (circled in Figure 2A) revealed the presence of few bacteria aggregated into small monolayer islands (Figure 3G and 3H).

In conclusion, the data so far presented indicate that a large proportion of GBS isolates can grow in biofilm under the experimental conditions used in this study, and that the capacity to form biofilms seems to be associated to the presence of type 2a pili on the bacterial surface.

### Pilus Variant 2a Is Specifically Required for Biofilm Formation *In Vitro*

**Figure 4:**
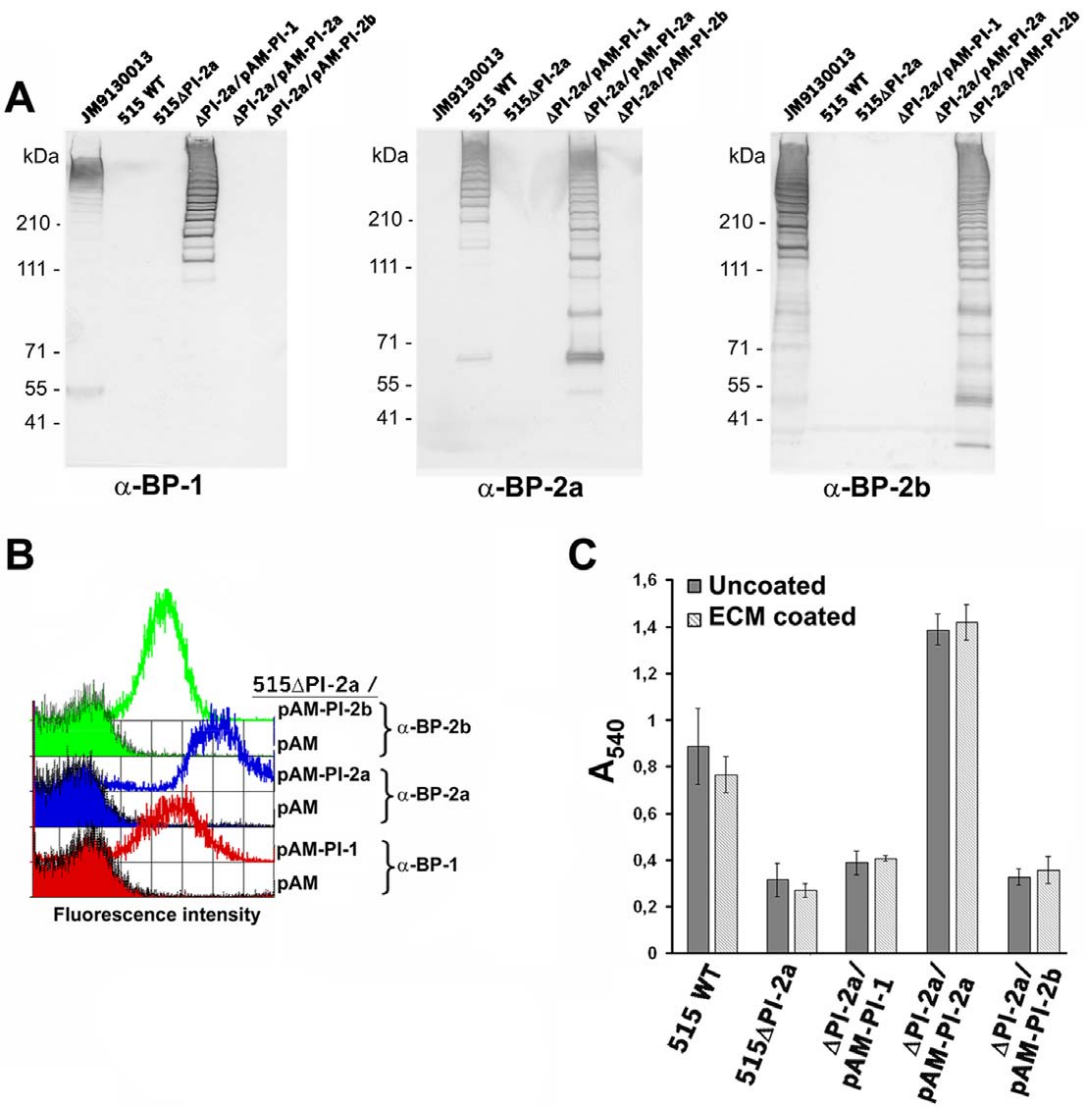
Role of the three pilus islands in biofilm formation. (A) Western Blot analysis of total protein extracts from GBS strain JM9130013 expressing both PI-1 and PI-2b, strain 515 expressing only PI-2a, the deletion strain 515ΔPI-2a (no pilus expression) and the strain 515ΔPI-2a, complemented with each of the three pilus islands: pilus island 1 (ΔPI-2a/pAM-PI-1), pilus island 2a (ΔPI-2a/pAM-PI-2a) and pilus island 2b (ΔPI-2a/pAM-PI-2b). The membranes were probed with antisera specific for the backbone proteins of pilus 1 (α -BP-1), pilus 2a (α -BP-2a) and pilus 2b (α -BP-2b). High molecular weight polymers, which indicate incorporation of the protein into pilus-like structures were detected. (B) Flow cytometry analysis of 515ΔPI-2a (no pilus expression), complemented with an empty pAM plasmid and with each of the three pilus islands, 515ΔPI-2a/pAM-PI-1, 515ΔPI-2a/pAM-PI-2a and 515ΔPI-2a/pAM-PI-2b, probed with antisera specific for pilus 1 (α -BP-1), for pilus 2a (α -BP-2a) and for pilus 2b (α -BP-2b). (C) Quantification of biofilm formation by crystal violet assay of GBS wild type strain 515 (515 WT), GBS 515ΔPI-2a and the complemented strains 515 ΔPI-2a/pAM-PI-1, ΔPI-2a/pAM-PI-2a and ΔPI-2a/pAM-PI-2b. Bacteria were grown under static conditions at 37°C for 18 h in 96-well uncoated polystyrene plates and on plates coated with a purified Extracellular Matrix (ECM) derived from human placenta. Crystal violet-stained, surface-attached cells were quantified by solubilizing the dye in 30% acetic acid and determining the absorbance at 540 nm. The mean values of three independent experiments and standard deviation are shown.

**Figure 5:**
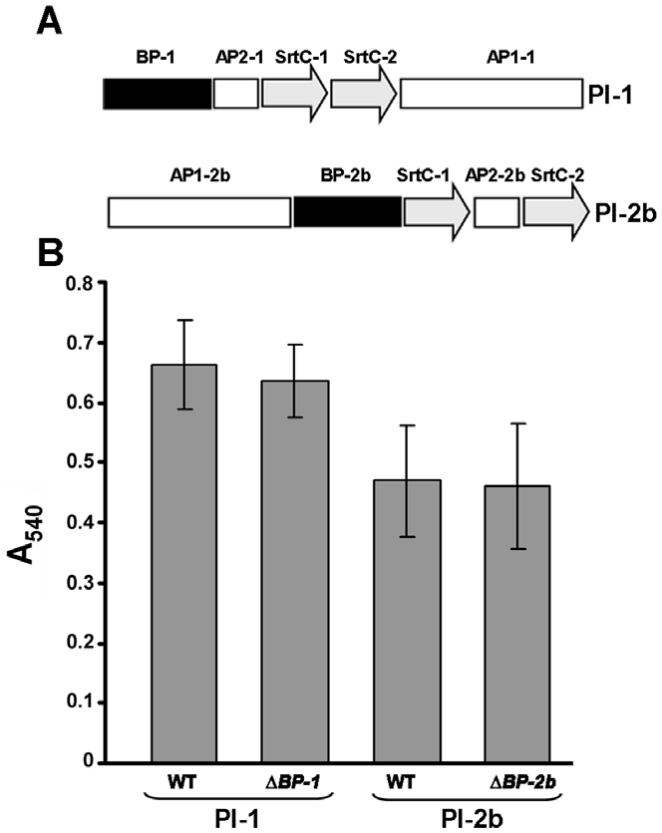
Role of pilus type 1 and pilus type 2b in biofilm formation. (A) Schematic representation of Pilus Island 1 (PI-1) and Pilus Island 2b (PI-2b). Genes encoding the three LPXTG proteins are represented by black (BP-1, backbone protein of pilus 1; BP-2b, backbone protein of pilus 2b) and white (AP1-1 and AP2-1, ancillary protein 1 and ancillary protein 2 of pilus 1; AP1-2b and AP2-2b, ancillary protein 1 and ancillary protein 2 of pilus 2b) arrows. SrtC transpeptidase genes (SrtC1 and SrtC-2) are shown in gray. (B) Quantification of biofilm formation by crystal violet assay of GBS wild type strain CJB111 expressing pilus type 1 (WT, PI-1) and GBS wild type strain ABC020017623 expressing pilus type 2b (WT, PI-2b) in comparison with the corresponding deletion mutant strains lacking the pilus expression. *ΔBP-1*, strain CJB111 with in-frame deletion of the gene coding for the backbone protein of pilus 1; *ΔBP-2b*, strain ABC020017623 with in-frame deletion of the gene coding for the backbone protein of pilus 2b. Bacteria were grown under static conditions at 37°C for 18 h in 96-well uncoated polystyrene plates and surface-attached, crystal violet-stained cells were quantified by solubilizing the dye in 30% acetic acid and determining the absorbance at 540 nm. The mean values of three independent experiments and standard deviation are shown.

The data shown above suggest that only pilus type 2a and not the other pilus types plays a role in biofilm formation. To support this finding, we generated an isogenic knock-out (KO) mutant strain 515ΔPI-2a in which the whole pilus island 2a was deleted and we complemented this non piliated strain with each of the three pilus islands identified in GBS. Complementation was carried out by transforming the mutant 515ΔPI-2a with recombinant pAM401 plasmids carrying the whole pilus islands. Western Blotting and FACS analyses using antibodies specific for each pilus type confirmed the absence of pili in 515ΔPI-2a and the expression of the corresponding pilus type in the complemented strains 515ΔPI-2a/pAM-PI-1, 515ΔPI-2a/pAM-PI-2a and 515ΔPI-2a/pAM-PI-2b (Figure 4A and 4B). The deletion mutant strain 515ΔPI-2a and its complemented strains expressing a single pilus type were then analyzed for their capacity to form biofilms on abiotic surfaces. As shown in Figure 4C, deletion of the whole pilus island 2a abolished the ability of bacteria to adhere to the plates and only the complemented strain expressing pilus 2a, and not those expressing pilus 1 and 2b, was able to significantly adhere to plates. Similar data were obtained when plates were coated with a purified Extracellular Matrix derived from human placenta, enriched of laminin, collagen type IV and heparan sulfate proteoglycan (Figure 4C). To further confirm the specific involvement of only pilus 2a in biofilm formation we investigated the ability to adhere to polystyrene plates of biofilm-forming strains expressing the other two pilus types (pilus 1 or pilus 2b), in which we knocked out the corresponding backbone protein essential for pilus polymerization (Figure 5A). These deletion mutant strains were not able to express pili on their surface (data not shown), yet the lack of pilus expression did not impact their ability to form biofilms on polystyrene plates (Figure 5B).

### Role of Pilus 2a Subunits and Sortase A in Biofilm Formation

**Figure 6:**
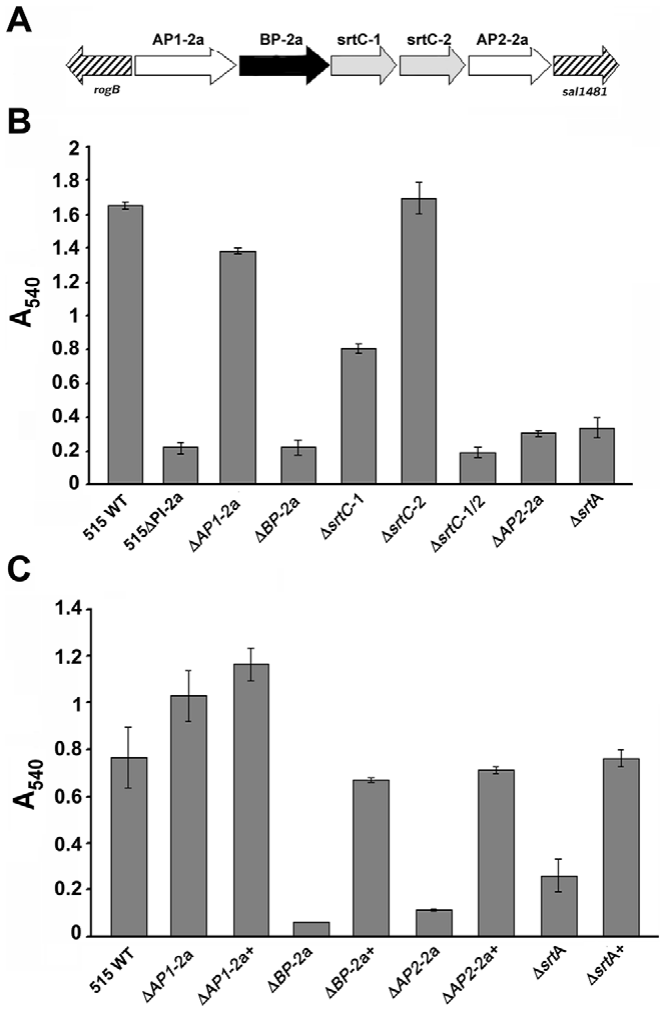
Contribution of pilus 2a subunits and sortase A in biofilm formation. (A) Schematic representation of pilus island 2a in GBS strain 515. Genes encoding the three LPXTG proteins are represented by black (backbone protein, BP-2a) and white (ancillary proteins 1 and 2, AP1-2a and AP2-2a) arrows. SrtC transpeptidase genes (SrtC1 and SrtC-2) are shown in gray. (B) Comparison of biofilm formation by crystal violet assay between GBS wild type strain 515 (515 WT), and the deletion mutant strains 515 for the whole pilus island 2a (515ΔPI-2a), for *AP1* gene (*ΔAP1-2a*), for *BP* gene (*ΔBP-2a*), for SrtC1 gene (*ΔsrtC1*), for SrtC*-2* gene (*ΔsrtC-2*), for both sortases genes (*ΔsrtC-1/2*), for *AP2* gene (*ΔAP2-2a*) and for Sortase A gene (*ΔsrtA*). Cells were grown in polystyrene plates at 37°C for 18 hours. Adherent bacteria were stained with crystal violet and quantified by measuring the absorbance at 540 nm. (C) Comparison of biofilm formation by crystal violet assay between the 515 deletion mutant strains (*ΔAP1-2a*, *ΔBP-2a*, *ΔAP2-2a*, *ΔsrtA*), complemented with an empty pAM401 expression vector alone and the corresponding complemented strains: *ΔAP1-2a+*, strain 515 *ΔAP1-2a* complemented by a plasmid containing the complete *AP1-2a* coding sequence; *ΔBP-2a+*, strain 515 *ΔBP-2a* complemented by a plasmid containing the *BP-2a* gene; *ΔAP2-2a+*, strain 515 Δ*AP2-2a* complemented by a plasmid containing the *AP2-2a* gene; *ΔsrtA*+, strain 515 *ΔsrtA* complemented by a plasmid containing the complete *srtA* coding sequence. Bacteria were grown under static conditions in polystyrene plates at 37°C for 48 h. Crystal violet-stained, surface-attached cells were quantified by solubilizing the dye in 30% acid acetic and determining the absorbance at 540 nm. The mean values of three independent experiments and standard deviations are shown.

To investigate the role of each gene of pilus island 2a (Figure 6A) in biofilm formation, we also analyzed biofilm formation in the mutant strains, generated in strain 515, lacking either one of the three structural LPXTG proteins (BP, AP1 and AP2) or the two SrtC-like sortases SrtC-1 and SrtC-2 [22]. As published elsewhere, inactivation of either the backbone protein BP or both sortases results in the loss of pilus whereas pilus assembly still occurs in mutants lacking the two ancillary proteins (AP1 and AP2) and one of the two sortases [22]. Consistently with these data, biofilm formation in mutants lacking pili was abolished (Figure 6B). By contrast, deletion of the *AP1* gene (*ΔAP1*), or deletion of the sortase SrtC-2 (*ΔsrtC-2*) involved in the incorporation of AP1 protein into the pilus structure, did not affect biofilm formation. Finally, the mutant lacking the *AP2* gene (*ΔAP2-2a*), as well as inactivation of sortase SrtC-1 (*ΔsrtC-1*), essential for AP2 incorporation into the pilus, reduced the ability of GBS to form biofilms (Figure 6B). Complementation of all biofilm-defective mutant strains restored biofilm formation to a level comparable with that of the wild-type strain (Figure 6C). Identical results were obtained both on abiotic and biotic surfaces (data not shown). The fact that mutant strains impaired in AP2 synthesis (*ΔAP2*) or AP2 covalent binding to the backbone protein BP (*ΔsrtC-1*) are unable to form biofilms is consistent with the role of the minor ancillary as pilus anchoring protein [28]. In terms of biofilm formation these mutants, which tend to release polymerized pili into the culture supernatants, behave like a non piliated strain. Also in line with this is the phenotype of *ΔsrtA* mutant. The constitutive SrtA catalyzes the covalent binding of the minor ancillary AP2 to peptidoglycan and since its inactivation leads to pilus secretion [28], the 515 *ΔsrtA* mutant is incapable of forming biofilms according to data obtained in GBS NEM316 *ΔsrtA* mutant strain (Figure 6B and [9]). The wild type phenotype was completely restored when the 515 *ΔsrtA* mutant strain was complemented with a plasmid expressing the complete *srtA* gene (*ΔsrtA*+) (Figure 6C).

**Figure 7:**
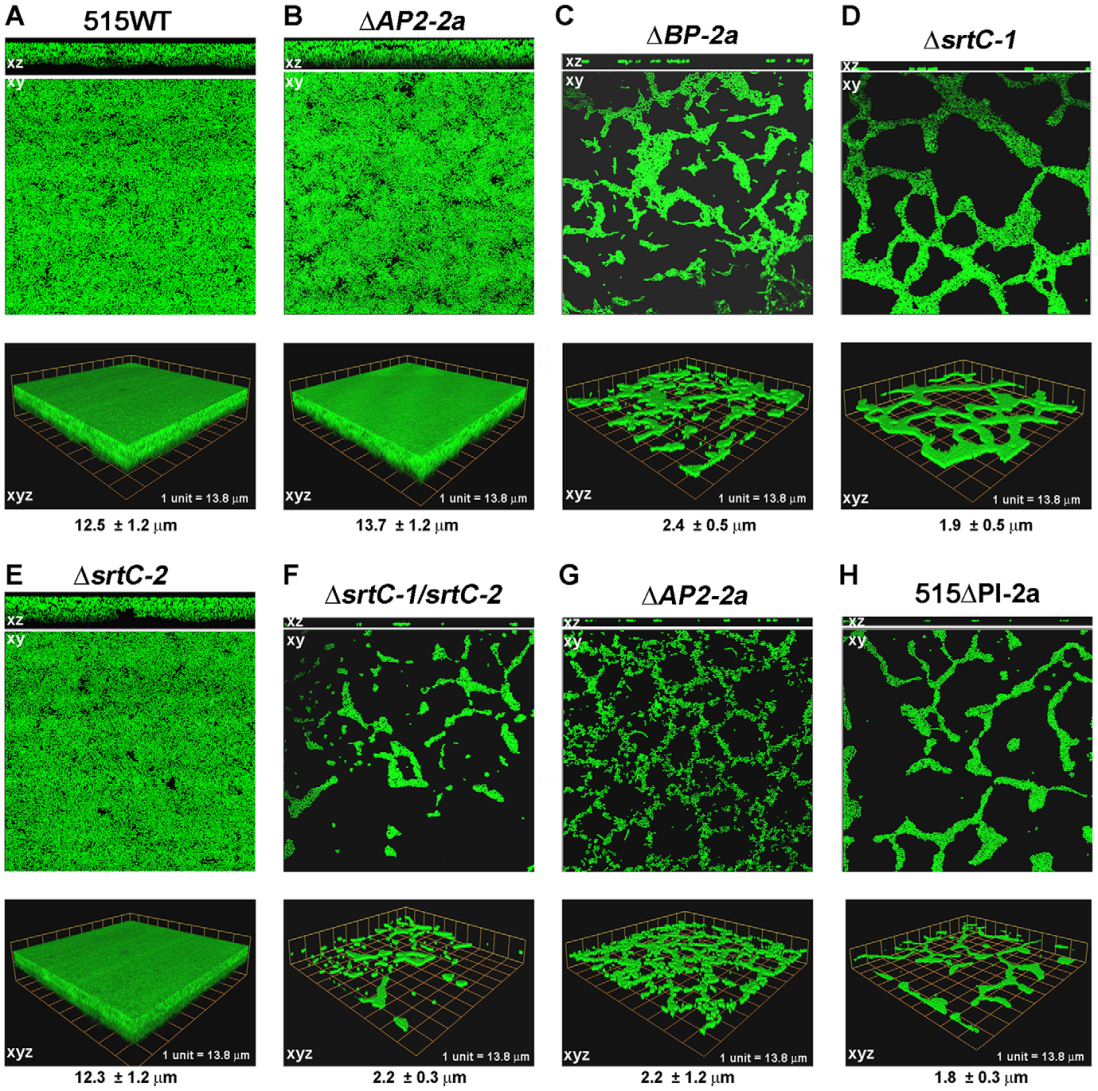
Confocal Laser Scanning Microscopy of biofilm formation by PI-2a deletion mutants. CLSM micrographs comparing biofilm formation of the wild type GBS strain 515 and the deletion mutant strains for each pilus 2a gene. (A) 515 WT, wild type strain 515; (B) *ΔAP1-2a*, strain 515 *ΔAP1-2a* with in-frame deletion of the *AP1-2a* gene; (C) *ΔBP-2a*, strain 515 *ΔBP-2a* with in-frame deletion of the *BP-2a* gene; (D) *ΔsrtC-1*, strain 515 *ΔsrtC-1* with in-frame deletion of the sortase *srtC-1* gene; (E) *ΔsrtC-2*, strain 515 *ΔsrtC-2* with in-frame deletion of the sortase *srtC-2* gene; (F) *ΔsrtC-1/2*, strain 515 *ΔsrtC-1/2* with in-frame deletion of both sortase genes; (G) *ΔAP2-2a*, strain 515 *ΔAP2-2a* with in-frame deletion of the *AP2-2a* gene; (H) 515ΔPI-2a, strain carrying deletion of the whole pilus island 2a. Bacteria were grown under static conditions at 37°C for 72 hours on glass coverslips. Then, biofilms were fixed and stained immediately prior to analysis with SYTO-9 (magnification 60×). Biofilm thickness was measured in different points of each field, and the means and standard deviations of at least three measurements are shown.

To further confirm the phenotypes observed in the CV assay, the entire panel of PI-2a deletion mutants was analyzed by confocal laser scanning microscopy (CLSM). Bacteria were incubated in chambers containing glass polylysine-coated coverslips and after 72 h the wells were emptied and washed. Adherent bacteria were stained with Syto-9 and examined by CLSM. Images were analyzed for morphology and biofilm thickness. As shown in Figure 7A, the wild type strain 515 formed structured multilayered aggregates of surface-adherent bacteria resembling a mature biofilm, with an average thickness of 12.5±1.2 µm. Similar structures were also seen with deletion mutants for AP1 and AP1-specific sortase SrtC-2, with an average thickness of 13.7±1.2 and 12.3±1.2 µm, respectively (Figure 7B and 7E). On the contrary, deletion mutants for the minor ancillary protein AP2 and the AP2-specific sortase SrtC-1 were biofilm-defective (Figure 7G and 7D). In addition, the double sortases mutant (Figure 7F), the deletion mutant for the backbone protein BP (Figure 7C), and the mutant lacking the entire pilus island (Figure 7H) were also ineffective in biofilm formation. Overall, all biofilm-defective strains formed a low-profile biofilm, averaging 2.3 µm in height.

Taken together, these data indicated that the presence of a polymerized pilus, correctly anchored to the cell wall, a process that involves both ancillary protein AP2, sortase SrtC-1 and Sortase A, is necessary for efficient biofilm formation. By contrast, loss of the major ancillary protein AP1 or of the sortase SrtC-2 responsible for AP1 incorporation in the pilus does not affect the ability of bacteria to adhere to surfaces.

### Antibodies against Surface-Exposed Pilus 2a Proteins Inhibit Biofilm Formation

**Figure 8:**
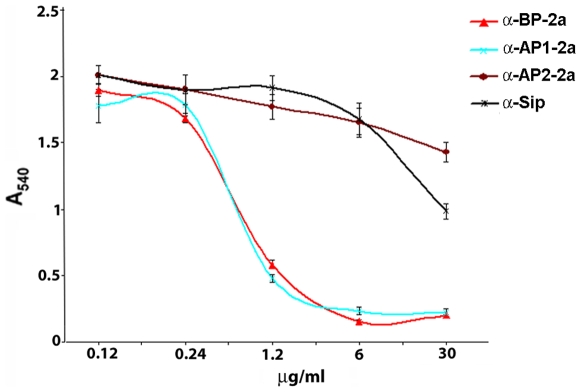
Inhibition of biofilm formation by antibodies against pilus 2a proteins. GBS strain 515 was grown in 96-well polystyrene plates at 37°C for 18 hours in the presence of serial dilutions of purified rabbit polyclonal antibodies directed against each of the pilus 2a structural protein (anti-BP-2a, anti-AP1-2a, anti-AP2-2a) and against the surface protein Sip (anti-Sip) used as a control. Biofilm formation was then measured by crystal violet assay, determining the absorbance at 540 nm. The mean values of three independent experiments and standard deviations are shown.

In order to obtain further insight into the role of pilus variant 2a in biofilm development, we investigated whether polyclonal antibodies against each of the three structural pilus proteins (BP, AP1 and AP2) inhibited the ability of GBS wild type strain 515 to form biofilms. Bacteria were grown under static conditions in the presence of increasing concentrations of purified polyclonal antibodies and biofilm was quantified after 18 hours. As reported in Figure 8, the presence of anti-BP antibodies significantly inhibited biofilm formation in a dose-dependent manner. Inhibition was also observed in the presence of anti-AP1 antibodies despite the fact that our deletion analysis showed that this ancillary protein is dispensable for biofilm formation. This result suggests that antibody binding to AP1, which is intimately associated to the backbone protein BP, is sufficient to interfere with the cell-to-cell contact promoted by pilus 2a. Finally, antibodies against AP2 had no effect on biofilm formation, in agreement with the location of this ancillary protein which, being embedded in the cell wall, is not accessible to antibodies [22], [28]. Biofilm inhibitory activity was pilus–specific since biofilm formation was unaffected in the presence of antibodies specific for the highly surface exposed protein Sip (TIGR annotation SAG_0032) [33]. Interestingly, antibodies specific for the backbone protein of pilus type 1 and 2b did not inhibit the capacity to adhere to plates of high-biofilm forming strains expressing on their surface at very high levels or pilus 1 or pilus 2b (data not shown). These data represent a further evidence that only pilus 2a, but not the other two pili, is involved in biofilm formation in vitro.

## Discussion

In pathogenic bacteria persistent colonization usually involves biofilm formation and pili have been shown to be part of the components which promote bacterial aggregation and attachment to the host surfaces. Since asymptomatic colonization of maternal vaginal mucosa by GBS is the major risk factor for sepsis and meningitis in newborns, and since GBS is decorated with pili, it was of interest to investigate the possible role of pili in GBS colonization and biofilm formation. We have previously shown that three pilus types (type 1, type 2a and type 2b), encoded by three distinct genetic islands, are present in GBS, and all isolates so far characterized carry one or a combination of two islands. Pilus type 2a, which includes seven variants based on the amino acid sequences of its structural proteins, appears to be the most represented of the pilus types, being present in over 70% of the clinical isolates analyzed and, in contrast with the other two pilus types, was found alone in a large number of strains (27%) [23]. Therefore, we first analyzed the ability of a type 2a-carrying strain (GBS 515) to form biofilms on both plastic and biotic surfaces and we demonstrated that the strain was in fact capable of growing as a surface-attached multicellular community resembling a biofilm-like structure. This is in line with data recently published by Konto-Ghiorghi and coauthors, who reported that GBS strain NEM316, also carrying type 2a pili, but belonging to a different variant, was able to form biofilms in microtiter plates [9]. Next, we characterized the biofilm-forming phenotype of a large panel of clinical isolates and we found that the majority of the biofilm formers carried type 2a pili. This observation led us to demonstrate by gene deletion/complementation analyses, that only type 2a pili, and not type 1 and type 2b, participate to biofilm formation in GBS. This is of particular interest since it is the first indication that the three pilus types in GBS fulfill different functions and that a particular pilus pattern is associated with specific functional features that could be relevant for pathogenesis. However, it remains to be clarified the biological significance of this difference. In fact, from our previous extensive analysis of pili distribution in 289 clinical isolates no significant correlation between pilus type and the nature/severity of disease emerged [23]. This apparent discrepancy, which deserves additional studies, could be explained considering that our pilus expression analysis and biofilm studies have been carried out under in vitro conditions which do not necessarily reflect the real situation during natural colonization/infection. Alternatively, or in addition, it becomes apparent that each pathogenic bacterial species is armed with a battery of different components/virulent factors which can be expressed in different combinations to escape the innate and adaptive immune responses of the host. In GBS, pili appear to be part of these virulent factors and the absence of type 2a pili in one isolate could be compensated by the expression of another, still to be identified, component that exerts similar functions (i.e. adhesion properties). Biofilm formation is a typical example of how bacteria utilize different components to reach the same goal, namely, colonizing the host mucosal surfaces. The multi-factorial process of cell-to-cell contact and adhesion has been demonstrated in a number of species, including Group A *Streptococcus* [8], [34], [35], [36], and, as shown in this work, it appears to be the case for GBS as well. Indeed, we observed that a quite consistent number of strains not expressing pilus 2a were able to form biofilms. Therefore, in these strains other still unknown factor(s) should be expressed to compensate for the absence of pili.

When we analyzed the role of each gene located in pilus island 2a in biofilm formation by gene deletion/complementation analysis, we found that any mutation that either abrogates pilus expression (deletion of the backbone protein, or concomitant deletion of sortase-encoding genes) or impairs pilus anchoring to the cell wall (deletions of *AP2*, *srtC-1*, *srtA* genes) prevents biofilm formation. On the contrary, inactivation of the major ancillary protein AP1, which decorates the shaft of type 2a pili [22], as well as the loss of the AP1-specific sortase-C (SrtC-2), does not affect the capacity of GBS strain 515 to adhere and aggregate on plastic. These data suggest that a polymerized pilus, correctly anchored to the cell wall, is essential for biofilm formation whereas the abrogation of AP1 expression does not impact both pilus polymerization and biofilm formation. Biofilm inhibition observed in the presence of antibodies specific for AP1, in contrast with the data for the AP1 mutant in biofilm development, may be explained as a steric hindrance resulting from antibody binding to AP1, which is intimately associated with the backbone protein BP. Conversely, the lack of inhibition of antibodies against the minor ancillary protein AP2 was expected, being this protein not surface-exposed, so not accessible to antibodies, as shown from previous works [22], [28]. However, the biofilm-defective phenotype shown by the mutant strain for the minor ancillary AP2 is consistent with the well demonstrated role of this protein as the anchor protein utilized by sortase A in covalent anchoring of pilus 2a to the cell wall [28]. According to this, also the sortase A mutant strain showed a defective phenotype as well as the KO strain lacking the sortase C specific for AP2 incorporation into pilus. Nevertheless our findings are partially in contrast with those reported for GBS strain NEM316 by Konto-Ghiorghi and coauthors. In this study the authors observed that deletion of the entire PilA gene (homologous of AP1 in GBS 515) caused loss of biofilm formation, while the deletion of PilC gene (homologous of AP2 in GBS 515) did not affect biofilm formation [9]. At present, the reason for this discrepancy is not clear. One possible explanation is that GBS 515 and GBS NEM316 carry type 2a pili belonging to different variants [23] whose structural components might differ in their role in cell aggregation and surface adhesion. In particular it would be interesting to investigate the role of PilC, which according to the data reported by Konto-Ghiorghi and coauthors, should not have the pilus anchoring role of its homologous AP2-2a [9], [28] and, differently from AP2-2a, should be exposed on the bacterial surface.

All three pili identified in GBS elicit protective immunity and vaccines based on a combination of pili components provide broad protection in the mouse model [23]. It is noteworthy that antibodies raised against both the backbone protein and the major ancillary protein of pilus island 2a block the formation of biofilms on both abiotic and biotic surfaces. These observations suggest that a pilus-based vaccine may work according to two different mechanisms of action, namely, the elicitation of opsonophagocytic antibodies capable of killing bacteria in a complement-dependent manner and the induction of antibodies capable of preventing colonization in the host. This observation could be of relevance for other piliated Gram-positive pathogens, including *S. pyogenes* and *S. pneumoniae*, known to grow in biofilm on biological surfaces and implanted medical devices such as catheters and artificial prosthetics [37].

## Materials and Methods

### Bacterial Strains, Plasmids, Media and Growth Conditions

GBS isolates analyzed in this work were collected by the Center for Disease Control and Prevention (CDC), Atlanta (100 strains); Baylor College of Medicine (BCM), Houston (103 strains) and Istituto Superiore di Sanità, Italy (86 strains). The classification of the strains on the basis of disease and serotype has been previously reported [23]. Strains CJB111 (type V), JM9130013 (type VIII) and 515 (type Ia) were kindly provided from Dr. Dennis Kasper (Harvard Medical School, Boston, MA, USA) [29]. The construction of the GBS 515 deletion mutant strains for each of the three structural LPXTG proteins of pilus type 2a (*ΔBP-2a*, *ΔAP1-2a*, Δ*AP2-2a*), for the two SrtC-family sortases, *ΔsrtC-1, ΔsrtC-2 and ΔsrtC-1/2* and for sortase A (*ΔsrtA*) have been already described [22], [28]. The in-frame deletion mutants for the entire pilus island 2a in 515 strain (515ΔPI-2a), for the backbone protein of pilus 1 in CJB111 strain (ΔBP-1) and for the backbone protein of pilus 2b in strain ABC020017623 (ΔBP-2b) were generated using Splicing by Overlap Extension (SOE) PCR as described previously [22], [38]. For the construction of 515ΔPI-2a, two DNA fragments were obtained by PCR amplification from genomic DNA using primers PI-2a-for/PI-2a-revA (5′-GATTTTGCTCTCGAGGCTTACCTTGAAAAATCTGCTGATG-3′/5′-CCAAGAATTGTCAGTCCTCCACCAACCATTTTCCGGTACGGTACTTTC-3′) and PI-2a-forA/PI-2a-rev (5′- GAAAGTACCGTACCGGAAAATGGTTGGTGGAGGACTGACAATTCTTGG-3′/5′- AGCTTCTGGCTCGAGTATAGCATCCCCTGAACCAGAAAC-3′). These fragments, overlapping for 24 bp, were used as a template to generate a PCR fragment comprising the flanking sequences of the PI-2a genomic locus using primers PI-2a-for/PI-2a-rev. For the construction of the mutant ΔBP-1 we used the primers BP-1-forA/BP-1-revA (5′- TCAGGATCCGCAACAATCTGTTCTGAC-3′/′5′- AACAGCAAAAGCCATCACTTTCGCTGGGCGTTCTTGTGACAC-3) and primers BP-1-forB/BP-1-revB (5′-CAAGAACGCCCAGCGAAAGTGATGGCTTTTGCTGTTAAGGGG-3′/5′-CGGGATCCAAATGCCAAAACAAGGAAACCCGC-3′). For the construction of the deletion mutant strain ΔBP-2b the primers used were: BP-2b-forA/BP-2b-revA (5′-TCAGGATCCGGTAACGGTGGTACTAGAGTAG-3′/5′- ACCTGTTGAAGGCAACTCAGTACCATCTTGAACTGTAATTGTCCCTGTC-3′) and BP-2b-forB/BP-2b-revB (5′-GACAGGGACAATTACAGTTCAAGATGGTACTGAGTTGCCTTCAACAGGT-3′/5′- GTGTGAGTCGACGCTTGTGCTACTTGGTAAACAAG-3′). The PCR deletion constructs were digested with the appropriate restriction enzyme (BamHI, XhoI or SalI) and ligated to the temperature-sensitive allelic exchange vector pJRS233, which was a gift from June Scott [39]. Transformations and allelic exchanges were performed essentially as described [40]. Screening for the deletion alleles was done by PCR amplification and sequencing using primers ΔPI-2a-for and ΔPI-2a-rev (5′-AAAGGGGTGAGTAAGGGACAAC-3′ and 5′-AACCTTTGTCTTCTCCCATCC-3′); primers ΔBP-1-for and ΔBP-1-rev (5′-AATGGTGGCGGGGTCAAC-3′ and 5′-TCTTTAGGGGCAATCTCCAACTG-3′) and primers ΔBP-2b-for and ΔBP-2b-rev (5′-CAGTAAAGGAACAGAATTGCC-3′ and 5′-CATAATCGCTCGAGATTGGTG-3′).

The construction of the complementation vectors 515 Δ*BP-2a+*, 515 Δ*AP1-2a+*, 515 ΔPI-2a/pAM-PI-1 and 515 Δ*srtA*+ have been previously described [22], [28], [41]. The complementation vector 515 Δ*AP2-2a+* was constructed using the primers p150-f (5′- CACCTGTCATGCGGCCGCGAAAGAGAAAGGGAAATCAAAA-3′) and p150-r (5′CTCTCTCTGAGATCTAACTTGCAATTGCAAGTT-3′) to amplify from the 515 genome a 1023 bp fragment containing the open reading frame coding for the AP2-2a protein. The complementation vectors 515ΔPI-2a/pAM-PI-2a and 515ΔPI-2a/pAM-PI-2b were constructed using the primers pPI-2a-f/pPI-2a-r (5′-CACCTGTCATGCGGCCGCCAATAGGAGTTGTAAAATGAG-3′/5′- CTCTCTCTGAGATCTCTAATTAGTAGTAGTG-3′) and primers pPI-2b-f/pPI-2b-r (5′- CACCTGTCATGCGGCCGCGGAGGATGCAAAAATGCTA-3′/5′-CTCTCTCTGAGATCTGTAGTAGATTGTATAATATTA -3′), respectively, to amplify from the strain 515 genome a 7753 bp fragment containing the entire pilus island 2a and from the strain COH1 genome a 8081 bp fragment comprising the entire pilus island 2b. All complementation vectors were electroporated into the corresponding deletion mutant strains. For all complementation experiments, the empty pAM401 plasmid, introduced into wild-type strain 515 and deletion mutant strains, was used as a negative control.

For PCR amplification experiments, genomic DNA was isolated from GBS strains by mutanolysin-treatment of bacterial cells using a NucleoSpin Tissue kit (Macherey-Nagel), according to the manufacturer's instructions. Plasmids and PCR products were purified using a Wizard Plus SV Miniprep System and a Wizard SV Gel/PCR Clean-Up System (Promega).

Bacteria were routinely grown at 37°C in Todd Hewitt Broth (THB; Difco Laboratories) or in trypticase soy agar plates supplemented with 5% sheep blood. For biofilm experiments bacteria were grown in Todd Hewitt broth (THB) supplemented with 1% glucose in 96-well polystyrene tissue culture plates.

### Biofilm Formation Assay

The biofilm formation assay used in this study is based on the ability of bacteria to adhere and form biofilms on solid surfaces as previously reported [42]. All biofilm experiments were performed in 96-well polystyrene flat-bottom microtiter plates (Costar), uncoated or coated with 3 µg/well of human Extracellular Matrix (BD, Becton Dickinson, Cat. No. 354237) overnight at 4°C. GBS strains grown overnight in THB-1% glucose were diluted 1∶20 in fresh medium and 100 µl cultures were added to each well previously rinsed three times with PBS. Wells filled with growth medium were included as negative controls. Plates were incubated without shaking at 37°C for 18 h aerobically in 5% CO_2_. Complemented strains were grown without antibiotic selection for 48 h. Before biofilm quantification, growth of wild type and mutant strains was assessed by measuring the absorbance of cultures in the wells at 600 nm with a spectrophotometer. Media, including any unattached bacteria, were then decanted from the wells, and any remaining planktonic cells were removed by rinsing with ddH_2_O. Wells were air dried, and adherent bacteria were stained for 10 min with a 0.5% (wt/vol) solution of Crystal Violet. After rinsing with ddH_2_O, bound dye was released from stained cells using 30% glacial acetic acid. This allowed indirect measurement of biofilms formed on both the bottom and sides of the well. Biofilm formation was quantified by measuring absorbance of the solution at 540 nm with a microplate reader (Tecan, Infinite M200). Each assay was performed in triplicate and repeated three times. For the biofilm inhibition assay, rabbit antibodies were purified using protein G sepharose, according to the manufacturer's instructions (Ab Spin Trap, GE Healthcare) and tested for their ability to inhibit biofilm formation. A GBS overnight culture was diluted 1∶50 in fresh medium and inoculated into wells of polystyrene flat-bottom 96-well microtiter plate with serial dilutions of purified antibodies in a final volume of 250 µl. Plates were incubated at 37°C for 18 hours aerobically in 5% CO_2_. Samples with no addition of purified antibodies or with normal rabbit serum were included as controls. Biofilm quantification was performed as already described.

Statistical analysis of biofilm formation in the panel of 289 GBS strains was performed using R-2.9.0 software. Kruskal-Wallis and Beherens-Fisher tests were used for the non parametric multi-group comparisons.

### Recombinant Proteins and Polyclonal Antibody Production

Genes coding for LPXTG proteins were expressed as 6His-tagged fusion proteins and purified by affinity chromatography as previously reported [21], [22], [23], [43]. Purified recombinant GBS proteins were used for intraperitoneal immunization of CD-1 outbred mice or to immunize New Zealand rabbits (Charles River Laboratories, Calco Italy). As reported previously, antisera against each protein did not show cross-reactivity with other pilin proteins [22].

### Western Blotting Analysis

Mid-exponential phase cells were harvested, washed in PBS and resuspended in 50 mM Tris-HCl, containing 400 units of mutanolysin (Sigma). Bacterial suspensions were then incubated at 37°C for 2 hrs and cells lysed by three consecutive cycles of freeze-thawing. Cellular debris were removed by centrifugation at 13000 rpm for 10 minutes and protein concentration measured by Bio-Rad Protein assay (Hercules, CA). Protein extracts (20 µg) were separated on 3–8% NuPage Novex SDS-PAGE gels (Invitrogen) and electroblotted onto nitrocellulose membranes. After blocking, membranes were incubated for 1 hour at room temperature (RT) with mouse primary antibodies diluted 1∶500, washed twice in PBS-Tween and incubated for 1 hour with a rabbit anti-mouse horseradish peroxidase-conjugated secondary antibodies (Dako). Bands were then visualized using an Opti-4CN Substrate Kit (Bio-Rad).

### Flow Cytometry

Exponential phase bacterial cells were harvested, washed in PBS, resuspended in PBS containing 0.08% (wt/vol) paraformaldehyde and incubated for 1 h at 37°C. Fixed bacteria were then washed once with PBS, resuspended in Newborn Calf Serum (Sigma) and incubated for 20 min. at 25°C. The cells were then incubated for 1 hour at 4°C in pre-immune or immune sera, diluted 1∶200 in dilution buffer (PBS, 20% Newborn Calf Serum, 0.1% BSA). Cells were washed in PBS-01% BSA and incubated for a further 1 h at 4°C with a 1∶100 dilution of R-Phycoerythrin conjugated F(ab)2 goat anti-mouse IgG (Jackson ImmunoResearch Laboratories; Inc.),. After washing, cells were resuspended in PBS and analyzed with a FACS Calibur apparatus (Becton Dickinson, Franklin Lakes, NJ) using FlowJo Software (Tree Star, Ashland, OR).

### Confocal Laser Scanning Microscopy (CLSM)

For all microscopic experiments, biofilms were allowed to grow at 37°C in 5%CO_2_ for 72 hours on glass sterile coverslips (BD, Biocoat) placed in 24-well polystyrene cell culture plates (Costar). Samples were then fixed with paraformaldehyde in 100 mM phosphate buffer pH 7.4 for 20 min, washed with PBS and blocked with PBS 3% (w/v) BSA, 1% (w/v) saponin (blocking solution) for 15 min. Fixed bacteria on coverslips were stained with SYTO-9 (488 nm excitation, green emission) or with red-fluorescent propidium iodide nucleic acid stain 490 nm (Molecular Probes). Coverslips were then washed with PBS and ddH_2_O, mounted on glass slides with the ProLong® Gold antifade reagent (Molecular Probes) and viewed on a BioRad Radiance 2000 Scanning Laser Confocal Microscope (CLSM). Three-dimensional immunofluorescence images were reconstructed from 0.5-µm confocal optical sections by using VOLOCITY 3.6 (Improvision, Lexington, MA). The confocal optical sections from random fields were collapsed into single projections.

### Scanning Electron Microscopy (SEM)

Bacteria were let to attach to poly-L-lysinated coverslide and then fixed in 2.5% paraformaldehyde-2.5% glutaraldehyde in cacodylate sucrose buffer (0.1 M cacodylate, 0.1 M sucrose, 5 mM CaCl_2_, 5 mM MgCl_2_, pH 7.2) overnight at 4°C, rinsed in cacodylate buffer at pH 7.2 (3 changes for 30 min at 4°C) and then post-fixed in 1% osmium tetroxide in the same buffer for one hour at 4°C. Then they were dehydrated in an ascending series of alcohol (30%–100%) and critical point dried in a Balzer's apparatus equipped with a liquid CO_2_ inlet and metal shadowed in a gold sputtering unit equipped with an argon inlet. Specimens were examined in a Jeol JSM 5200 scanning electron microscope.
